# Laparoscopic Repair of Morgagni Hernia Combined with Right Hemicolectomy for Bleeding Ascending Colon Carcinoma Lodged within the Chest: A Case Report and Review of the Literature

**DOI:** 10.1155/2021/5533203

**Published:** 2021-07-19

**Authors:** Oluwatobi O Onafowokan, Kiran Khosa, Hugo Bonatti

**Affiliations:** Meritus Surgical Specialists, USA

## Abstract

**Background:**

Morgagni hernias are rare in adults and may be asymptomatic but, nevertheless, require surgical repair, with laparoscopy offering an excellent option. The colon dislodged into the chest through diaphragmatic hernias may be affected by various disorders, including malignancies. *Case Report*. A 70-year-old obese male presented with fatigue and shortness of breath. CT scan showed the right colon lodged in the chest through a Morgagni hernia. He was anaemic, and colonoscopy revealed a colon cancer. He underwent combined laparoscopic hernia repair with bioabsorbable mesh and right hemicolectomy. Recovery was uneventful, but the patient died 5 months later from chemotherapy-associated cardiac failure. Literature review revealed eight similar published cases, and including ours, there were seven Morgagni hernias, one traumatic hernia, and one Bochdalek hernia. Median age of the five men and four women was 66 (range 49-85) years. Surgical approach was thoracotomy (2), laparotomy (5), and laparoscopy (2).

**Conclusion:**

Outcome of the rare condition is determined by the course of the colon cancer. Hernia repair was successful in ours and all other published cases. A combined laparoscopic approach can be safely done.

## 1. Introduction

Diaphragmatic hernias may be congenital, such as Bochdalek and Morgagni hernias, or acquired, such as traumatic hernias [1, 2]. Morgagni hernia is less common than Bochdalek hernia. They are more frequent in women (female : male = 3 : 1), have a hernia sac, and may contain intraabdominal viscera; left-sided anterior hernias are extremely rare [3]. Adults with diaphragmatic hernias may remain asymptomatic for a prolonged time or present with nonspecific gastrointestinal symptoms such as right upper quadrant pain or bloating or with respiratory symptoms including chest pain and shortness of breath. These hernias may be incidentally found on imaging [4]. Hernia contents predominantly include the greater omentum and transverse colon; and less frequently the small intestine, liver, and stomach [4]. Bowel obstruction and perforation of intestine within the chest mandate emergent surgery. Due to the risk of visceral strangulation, elective surgical repair is recommended, even in asymptomatic patients [3].

Traditionally, hernia closure was done using laparotomy and thoracotomy; however, laparoscopy and thoracoscopy now offer a less invasive approach [2, 5]. Surgery involves reduction of herniated organs, removal of the hernia sac from the mediastinum to prevent recurrence [3], and closure of the defect with running or interrupted sutures, such as transfascial closure using a suture passer. Mesh reinforcement and interposition are options and depend on the size of the hernia.

If the colon is lodged within the chest through a diaphragmatic hernia, it may be affected by various pathologies such as colitis and diverticulitis, as well as colon polyps and colon cancer [6–14]. Laparoscopy has emerged as the preferred surgical approach for oncologic colectomy [15], which is followed by stage-dependent adjuvant chemotherapy.

We herein report a patient with a Morgagni hernia harboring a bleeding ascending colon adenocarcinoma, and also report data from a review of the literature on similar cases.

## 2. Case Presentation

**Figure 1:**
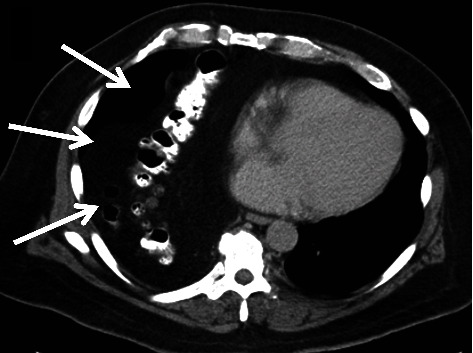
CT scan: colon and omentum lodged within the right chest (white arrows).

**Figure 2:**
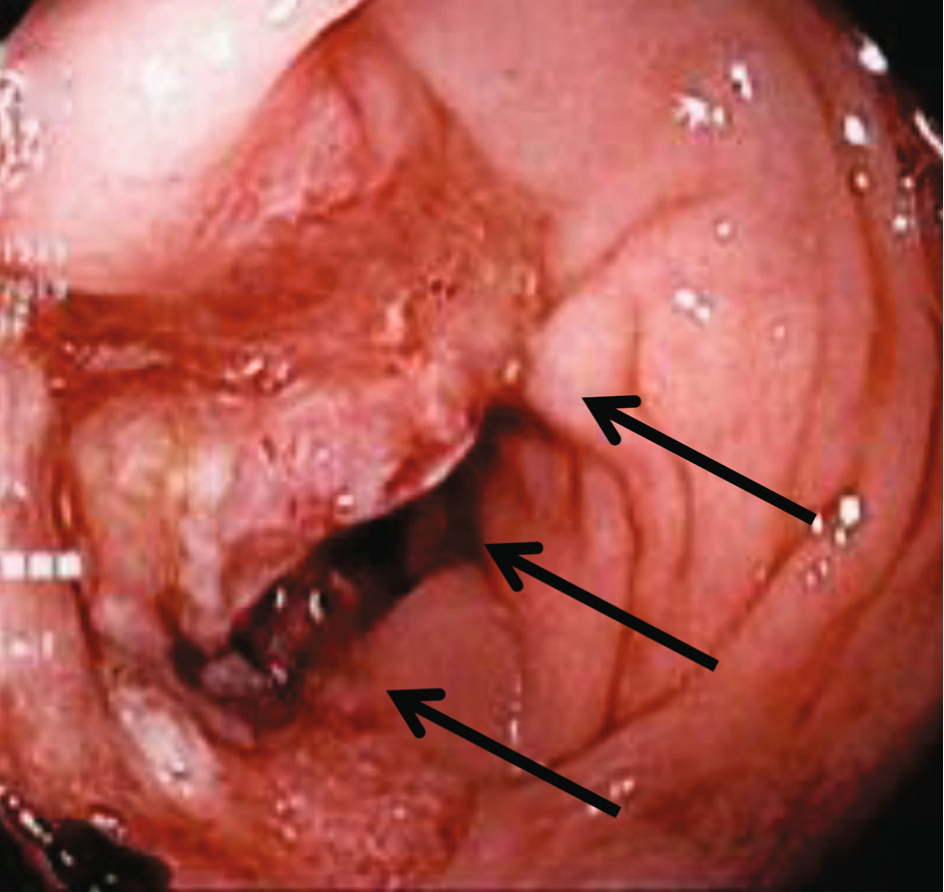
Colonoscopy (endoscope entered chest through the Morgagni hernia during exam). Mass in ascending colon (black arrows).

A 70-year-old obese male presented to the emergency room (ER) with fatigue and shortness of breath. He reported that during the past few weeks, he had developed progressive breathing difficulties and weakness. He had no fever and only mild right upper quadrant and right chest discomfort. On physical examination, diminished breath sounds in the right lower lung were found. The patient was anaemic (haemoglobin 8 mg/dl), and leukocyte count was normal. The CT scan showed a large Morgagni-type hernia with the entire right hemicolon, parts of the transverse colon and terminal ileum, and ample omentum trapped in the chest (Figure 1), causing atelectasis of the right lower lobe. After stabilization in the ER, he was admitted to the hospital, and workup for the blood loss anaemia was initiated. He had a positive stool guaiac test and underwent a colonoscopy showing a large mass in the right colon (Figure 2). During colonoscopy, the endoscope was maneuvered with extra care to avoid any injuries; the colonoscope entered the right chest but could ultimately be advance to the ileocecal valve. Biopsies of the mass in the proximal ascending colon revealed an adenocarcinoma.

**Figure 3:**
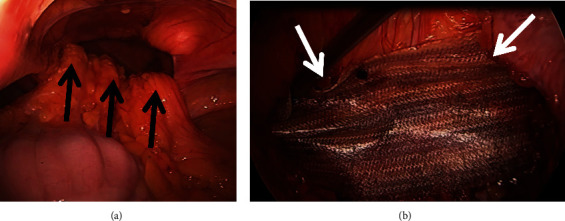
Intraoperative findings. (a) Large right anterior diaphragmatic defect: the contents are gently reduced (black arrows). (b) A Phasix Mesh (white arrows) is placed to reinforce the hernia closure.

He consented for laparoscopic repair of the diaphragmatic hernia and resection of the right colon. Surgery was done in the supine position and was initiated by insertion of a 5 mm 1^st^ entry port in the left upper quadrant. An additional 5 mm port was inserted in the left lower quadrant and a 10-12 mm port above the umbilicus. A large right-sided diaphragmatic defect (7 cm diameter) was encountered anteriorly to the liver with the entire right hemicolon and terminal ileum and omentum trapped inside. The hernia contents were gently reduced from the mediastinum (Figure 3(a)). The peritoneum at the anterior aspect of the defect was incised using an Enseal, and the entire hernia sac was carefully mobilized out of the mediastinum, avoiding injury to the pleura or phrenic nerve. The Valsalva maneuver was applied to protrude the hernia sac from the chest facilitating exposure. The anterior portion of the hernia sac was resected and placed into a retrieval bag. The hernia defect was closed with multiple transfascial sutures. As this was a clean contaminated case, a 10 × 7 cm bioabsorbable Phasix™ ST Mesh (Bard, Warwick, RI, USA) was placed to protect the reconstruction (Figure 3(b)); and the mesh was extraperitonealized using the dorsal flap of the hernia sac.

The redundant and partially twisted ascending colon was completely mobilized. The mesentery and omentum were thickened and chronically inflamed from displacement in the chest. The vascular pedicle was isolated and stapled. A 4 cm periumbilical incision including the 10-12 mm port site was made, and the colon was eviscerated and resected, with creation of an extracorporeal ileocolic anastomosis using two loads of the 75 mm GIA. The hernia sac was then removed from the abdomen. Pathology revealed pT3N1M0 moderately differentiated adenocarcinoma (4 cm diameter) with one of 50 harvested lymph nodes being positive.

The patient had no postoperative complications and was discharged after three days, with returned bowel function. A port-a-cath was placed two weeks later, and chemotherapy was initiated. He did well, and chest X-ray showed no evidence of a recurrent diaphragmatic hernia. He died five months later from a cardiac event possibly associated with chemotherapy.

## 3. Discussion

We herein report the first^st^ patient undergoing right hemicolectomy together with Morgagni hernia repair using a complete laparoscopic approach. This is only the second^nd^ case of a colon cancer in a Morgagni hernia in the US, and the seventh^th^ case worldwide.

A review of the literature was undertaken using PubMed and Google Scholar databases, with search criteria including: Morgagni hernia, Bochdalek hernia, diaphragmatic hernia, hiatal hernia, and paraesophageal hernia; combined with colon cancer/carcinoma. This revealed only eight similar patients. A case in which barium enema suggested colon cancer but intra-operatively no colon mass was found, was excluded [16]. Karakis et al. described a left colon cancer projecting in the chest due to left diaphragmatic eventration and not a diaphragmatic hernia [17].

Table 1 summarizes the previously reported eight similar cases and our case. The first case of colon cancer in Morgagni hernia was published in 1977 by Dawson and Jansing [7] from Kentucky, USA, within a series of Morgagni hernias. Doutre et al. [8] in 1980 in France and subsequently Kochling et al. in 1990 in Germany [10] reported the next cases. Kochling et al.'s patient had liver metastases at the time of diagnosis and received palliative chemotherapy. He had later an open ileocolonic bypass for bowel obstruction. The cancer was in the distal transverse colon, and the diaphragmatic defect was on the left side, therefore more likely being a Bochdalek or left Morgagni hernia. The next report from Norway described a patient with a chest mass and pleural empyema, which was drained, and after recovery, transthoracic surgery was done, but no operative details are provided [6]. A case from Greece described the only colon cancer in a traumatic left diaphragmatic hernia, which was approached through a thoracoabdominal incision [11]. Turner et al. from New Zealand reported an appendix adenocarcinoma in a Morgagni hernia, and they used a Chevron incision for access [13]. The last two cases came from Bosnia-Herzegovina and Spain [9, 12]; one patient underwent emergent laparotomy, and the other case by Rabal Fueyo published in 2018 was the first^st^ case with a laparoscopic approach of the hernia, but the colectomy was done in an open fashion. Outcome in the reported cases was determined by the course of the malignancy. The diaphragmatic hernia repair was done in most cases with interrupted sutures, but mesh repair was used in three patients and no recurrent diaphragmatic hernia was reported. In our case, transfascial absorbable sutures were used and the reconstruction was reinforced with a Phasix Mesh. This was done prior to the colon resection, and the mesh was covered with the posterior flap of the hernia sac to protect against infection. Morgagni hernia repair has in general a good prognosis, with low operative morbidity and mortality and low recurrence rates [4].

Intrathoracic colon cancers associated with diaphragmatic hernias are very rare occurrences. It is unclear why colon cancer in Morgagni hernias is much more common than in other diaphragmatic hernias. We have not found a single case of colon cancer associated with a type 4 hiatal hernia; however, large paraesophageal hernias have been found to contain gastric cancer [18] and other tumors such as gastrointestinal stromal tumors [19], with Wolfe et al. reporting ovarian cancer metastases causing a symptomatic paraesophageal hernia [20]. One explanation for the differences seems to be the much higher incidence of ascending colon compared to descending colon cancer. In addition, the right colon is rather mobile and can easily protrude through a Morgagni hernia. If the transverse colon contains a cancer, proximal colonic dilatation may push the colon through a preexisting right- rather than a left-sided diaphragmatic defect. Herniation of the sigmoid colon, which has the second^nd^ highest cancer rate in the colon, through the diaphragm is very unlikely. Development of a left-sided iatrogenic diaphragmatic hernia has been reported after laparoscopic resection of a splenic flexure cancer [21].

Our patient presented with anaemia, and colonoscopy established diagnosis of a colon cancer. Colonoscopy was done extremely carefully especially during passage through the chest. Only one of the previously reported patients had a colonoscopy as well; however, this patient's diagnosis had been already established by biopsy of his liver metastases [10].

To summarize, colon cancer in diaphragmatic hernias is extremely rare. Combined laparoscopic repair of the hernia and oncologic colectomy can be safely undertaken.

## Figures and Tables

**Table 1:** Results from review of the literature.

#	Authors	Year	Origin	Age	m/f	Type of hernia	Clinical presentation	Imaging	Colonoscopy	Surgical approach	Colectomy	Hernia repair	Comments	Outcome
1	Dawson RE	1977	KY, USA	67	f	Morgagni hernia	Malaise, anemia	Barium enema: obstructing colon mass within Morgagni hernia	nd	Midline laparotomy	Transverse colectomy	Primary closure	1st case, part of series of Morgagni hernia repairs	Uneventful recovery; had liver metastases at time of surgery
2	Doutre LP	1980	France	78	f	Morgagni hernia	Bowel obstruction	Barium enema: no tumor seen but Morgagni hernia	nd	Midline laparotomy	Right hemicolectomy	Primary closure	Published in French	Uneventful recovery
3	Kochling G	1990	Germany	51	m	Left Morgagni or Bochdalek hernia	Weight loss, diarrhea, leukocytosis	CT scan: Morgagni hernia, liver lesions	Mass in intrathoracic ascending colon	Palliative chemotherapy, secondary midline laparotomy	Palliative ileocolic anastomosis	NDA	Published in German; described as left chest Morgagni hernia; distal transverse colon cancer	Died from tumor progression after 3 months
4	Arslan A	2000	Norway	60	f	4 cm Morgagni hernia	Weight loss, epigastric pain, shortness of breath	CT scan: pleural empyema, 10 cm colon mass in Morgagni hernia	nd	Emergent pleural empyema drainage, then right thoracotomy	Segmental colectomy	Primary closure	Not in PubMed	NDA
5	Pappas-Gogos G	2007	Greece	66	m	Traumatic left diaphragmatic hernia	Left chest pain	CT scan: colon mass in traumatic diaphragmatic hernia	nd	Left thoracoabdominal incision	Left hemicolectomy	PTFE patch closure	Traumatic left-sided hernia; splenic flexure cancer	Uneventful recovery, well after two years
6	Turner G	2013	New Zealand	50	m	Morgagni hernia	Constipation, abdominal pain	CT scan: cecal mass in Morgagni hernia; PET: cecal FDG uptake	nd	Chevron incision	Right hemicolectomy	Primary closure	Appendix adenocarcinoma; Morgagni hernia known from cxr 5 years earlier	Uneventful recovery
7	Gaco S	2013	Bosnia & Herzegovina	85	m	7 cm Morgagni hernia	Acute colonic obstruction	CT scan: obstructed colon in Morgagni hernia, no mass	nd	Emergency midline laparotomy	Palliative ileocolic anastomosis (locally advanced cancer)	Primary closure	Emergent case	Discharged after 3 days, palliative chemotherapy; alive after 10 months
8	Rabal Fueyo A	2018	Spain	49	f	5 cm Morgagni hernia	Abdominal pain	CT scan: colon mass in Morgagni hernia	NDA	Laparoscopy	Extended right hemicolectomy through limited laparotomy	Nonabsorbable MESH closure	1st laparoscopic approach: only hernia repair	Discharged after 7 days; no FU data
9	Current case	2020	MD, USA	70	m	7 cm Morgagni hernia	Fatigue, SOB	CT scan: Morgagni hernia	Mass in intrathoracic ascending colon	Laparoscopy	Laparoscopic right hemicolectomy with extracorporeal anastomosis	Transfascial sutures; absorbable MESH and peritoneal flap	1st total laparoscopic case	Discharged after 5 days; died after 5 months from complications of chemotherapy

Abbreviations: m: male; f: female; nd: not done; NDA: no data available; KY: Kentucky; MD: Maryland; PTFE: polytetrafluoroethylene.

## Data Availability

The data cannot be shown due to HIPAA restrictions.
